# Editorial: Significant influencing factors and effective interventions of mobile phone addiction, volume II

**DOI:** 10.3389/fpsyg.2023.1269071

**Published:** 2023-08-24

**Authors:** Qingqi Liu, Zongkui Zhou, Christiane Eichenberg

**Affiliations:** ^1^College of Education for the Future, Beijing Normal University, Zhuhai, Guangdong, China; ^2^School of Psychology, Central China Normal University, Wuhan, China; ^3^Faculty of Medicine, Sigmund Freud University Vienna, Vienna, Austria

**Keywords:** mobile phone addiction, individual factors, environmental factors, interventions, mechanisms

The issue of mobile phone addiction has garnered significant attention among researchers over the past two decades. Expanding on our previous work, we have successfully completed the Research Topic titled “*Significant Influencing Factors and Effective Interventions of Mobile Phone Addiction - Volume II*”. This Research Topic encompasses both empirical research and meta-analysis studies. Empirical studies allow for the discovery of new insights, while meta-analysis enables the formulation of more robust conclusions based on a synthesis of multiple research findings. Meta-analyses in our Research Topic have extensively synthesized previous research findings regarding the impact of self-esteem, self-control, social support (Ding et al.), and academic burnout (Li S. et al.) on and exercise (Zhang et al.) mobile phone addiction. Our Research Topic has made notable advancements in understanding the causes of mobile phone addiction, as well as the practical implications for addressing this issue through effective interventions.

Our Research Topic contributes to a comprehensive understanding of influencing factors from both individual and environmental perspectives. Regarding individual factors, according to the Interaction of Person-Affect-Cognition-Execution model (Brand et al., 2019), addictive behaviors are the result of the interaction between individual personality, cognitive factors, emotional factors, and executive functions. Our Research Topic align well with the Person-Affect-Cognition-Execution model. In our Research Topic, personality factors such as self-esteem and self-control (Ding et al.), emotional factors such as anxiety and depression (Ge et al.), loneliness (Li G.-R. et al.), and academic burnout (Li S. et al.), cognitive factors such as automatic thoughts (Lian et al.), as well as executive dysfunction (Ge et al.), have all been confirmed to have significant predictive effects on mobile phone addiction. From an environmental perspective, the family is an important environmental micro-system that influences mobile phone addiction (Liu et al., 2020). In our Research Topic, family functioning (Li G.-R. et al.), family cohesion and adaptability (Lian et al.), and parenting style of encouraging autonomy (Li Z.-k. et al.) have all been found to be closely associated with mobile phone addiction. Moreover, based on comprehensive research findings, environmental factors not only predict mobile phone addiction through the mediation of individual factors, but also the predictive effect of environmental factors on mobile phone addiction varies depending on certain individual factors. In other words, mobile phone addiction is a complex outcome resulting from the joint influence of external environmental factors and internal individual factors.

Our Research Topic delves deeper into intervention methods and their effectiveness. One study examines the direct and indirect effects of physical exercise on Internet addiction (Cheng et al.). Another meta-analysis systematically summarizes and compares the intervention effects of different types of exercise on Internet addiction (Zhang et al.). These studies not only inspire further research on internet addiction/mobile phone addiction but also offer empirical support and practical recommendations for future educational practices. Indeed, there are numerous other efficacious intervention approaches to address internet addiction and mobile phone addiction, including cognitive-behavioral therapy (Kim et al., 2018), mindfulness intervention training (Li et al., 2018), and family therapy (Liu et al., 2015), among others. Integrating physical exercise with these interventions has the potential to augment their practical efficacy in everyday life.

In summarizing our two Research Topics, it is evident that they hold significant theoretical and practical implications. However, they also present several areas that merit further investigation by future researchers. Firstly, our Research Topics did not comprehensively explore the complex interactions between multiple environmental systems and various individual factors. To achieve research results of greater ecological validity, it is crucial to consider the combined effects of these factors. Secondly, our Research Topics lacked studies simultaneously analyze and compare the effects of certain influencing factors on different types of internet addiction/mobile phone addiction. Different types of internet addiction/mobile phone addiction exhibit distinct behavioral characteristics (Liu et al., 2022). Revealing the influencing patterns of different types of internet addiction/mobile phone addiction would contribute to more targeted interventions in practice. Lastly, our Research Topics did not extensively examine the intervention effects of various methods on mobile phone addiction, including cognitive-behavioral therapy, mindfulness intervention, family therapy, and compound psychotherapy. Attachment-based therapy may also be an effective intervention for mobile phone addiction, as insecure attachment has been identified as a risk factor (Eichenberg et al., 2019). In future research, it would be beneficial to focus on analyzing the effects, mechanisms and conditions of multiple intervention methods.

## Author contributions

QL: Writing—original draft, Writing—review and editing. ZZ: Writing—review and editing. CE: Writing—review and editing.
